# A Cross-Scale Spatial–Semantic Feature Aggregation Network for Strip Steel Surface Defect Detection

**DOI:** 10.3390/ma18245567

**Published:** 2025-12-11

**Authors:** Chenglong Xu, Yange Sun, Linlin Huang, Huaping Guo

**Affiliations:** School of Computer and Information Technology, Xinyang Normal University, Xinyang 464000, China; clxu@xynu.edu.cn (C.X.); linlinhuang11@163.com (L.H.)

**Keywords:** surface defect detection, feature aggregation, attention mechanism, adaptive RPN

## Abstract

Strip steel surface defect detection remains a challenging task due to the diverse scales and uneven spatial distribution of defects, which often lead to incomplete feature representation and missed detections in sparsely distributed regions. To address these challenges, we propose a novel cross-scale spatial–semantic feature aggregation network (CSSFAN) that achieves fine-grained and semantically consistent feature fusion across multiple scales. Specifically, CSSFAN adopts a bottom-up feature aggregation strategy equipped with a series of cross-scale spatial–semantic aggregation modules (CSSAMs). Each CSSAM first establishes a mapping relationship between high-level feature points and low-level feature regions and then introduces a cross-scale attention mechanism that adaptively injects spatial details from low-level features into high-level semantic representations. This aggregation strategy bridges the gap between spatial precision and semantic abstraction, enabling the network to capture subtle and irregular defect patterns. Furthermore, we introduce an adaptive region proposal network (ARPN) to cope with the uneven spatial distribution of defects. ARPN dynamically adjusts the number of region proposals according to the local feature complexity, ensuring that regions with dense or subtle defects receive more proposal attention, while sparse or background regions are adaptively suppressed, thereby enhancing the model’s sensitivity to defect-prone areas. Extensive experiments on two strip steel surface defect datasets demonstrate that our method significantly improves detection performance, validating its effectiveness and robustness.

## 1. Introduction

Strip steel serves as a fundamental material in the steel industry and is widely utilized in automotive manufacturing, construction, household appliances, and energy sectors, playing a critical role in industrial production and national economic development [1,2,3]. However, during production and storage, the surface of strip steel inevitably suffers from various defects caused by factors such as rolling, cooling, transportation, and environmental corrosion. Typical defects include scratches, pits, folds, spots, and cracks [4,5,6]. These defects not only degrade the surface quality and mechanical properties of strip steel but also can cause fracture, stress concentration, or even product scrapping in subsequent processing, ultimately reducing production efficiency and causing economic losses. Therefore, accurate detection of strip steel surface defects holds significant theoretical importance and practical value, particularly for ensuring product quality [7,8,9].

Early approaches to strip steel surface defect detection primarily relied on handcrafted features and conventional image processing techniques, including gray-level statistics, edge detection, and texture modeling [10,11,12]. While these methods are relatively simple to implement and require limited computational resources, they suffer from several inherent limitations [13]. Specifically, their effectiveness is highly dependent on expert prior knowledge and carefully designed feature descriptors, which restricts adaptability to new scenarios. Moreover, due to their shallow representational capacity, these approaches struggle to capture the complex appearance variations of defects caused by changes in illumination, surface roughness, and background noise [14]. As a result, they exhibit poor robustness and limited generalization capability, often failing to deliver reliable detection performance in real-world industrial environments where defect types are diverse and imaging conditions are unconstrained [15].

In recent years, deep learning techniques have achieved significant progress in the field of computer vision and have been increasingly applied to strip steel surface defect detection tasks [16,17,18]. Convolutional neural network (CNN)-based detection methods can automatically learn hierarchical feature representations, effectively overcoming the limitations of traditional handcrafted features [19]. To further improve the performance of CNN-based methods, researchers have proposed various improvement strategies. For instance, Sohag et al. [20] proposed an improved Faster R-CNN by integrating the Swin Transformer with a path aggregation feature pyramid network (PAFPN) [21], strengthening the model’s capability to capture complex defect. Han et al. [22] developed a scale-aware feature pyramid network that leverages an attention mechanism to reduce semantic discrepancies across pyramid levels, improving multiscale feature consistency. Hou et al. [23] designed a spatial attention encoder that establishes long-range dependencies to capture global contextual cues, enhancing the perception of defects. Similarly, Lu et al. [24] designed an anchor-free detector based on the AutoAssign framework, which enhances the semantic representation of defects while suppressing background interference.

**Figure 1:**
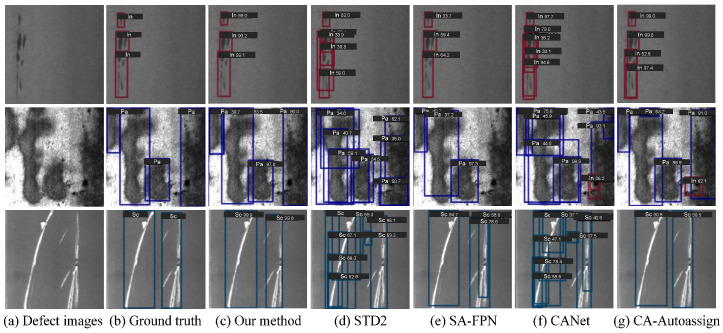
Comparison of detection results between our method and other state-of-the-art strip steel surface defect detection approaches.

Despite the remarkable progress achieved by the aforementioned CNN-based methods, challenges remain when dealing with defects of varying scales and irregular distributions, which often lead to incomplete feature representation and inaccurate localization in complex industrial scenarios. As shown in Figure 1, STD2 [20] generates a large number of redundant bounding boxes when handling defects with diverse shapes and textures, making it difficult to accurately distinguish adjacent regions. SA-FPN [22], limited by its ability to discriminate complex background features, tends to misclassify true defect regions as background, resulting in missed detections. CANet [23] struggles to differentiate small-scale defects from background textures, often misidentifying background patterns as defects, which leads to false positives. In addition, CA-Auoassign [24] excessively focuses on background noise, which introduces false detections.

To address the aforementioned challenges, we propose a novel cross-scale spatial–semantic feature aggregation network (CSSFAN) for steel surface defect detection. Specifically, CSSFAN adopts a bottom-up feature aggregation strategy equipped with a series of cross-scale spatial–semantic aggregation modules (CSSAMs). Each CSSAM first establishes a spatial correspondence between high-level feature points and low-level feature regions, and then introduces a cross-scale attention mechanism to adaptively inject spatial details from low-level features into high-level semantic representations. This design bridges the gap between spatial precision and semantic abstraction, enabling the network to capture subtle and irregular defect patterns that are often overlooked by conventional multiscale fusion methods. Furthermore, we introduce an adaptive region proposal network (ARPN) to handle the uneven spatial distribution of defects. Unlike traditional RPNs that use a fixed number of anchors across the entire image, ARPN dynamically adjusts the number of region proposals based on local feature complexity.

In summary, the main contributions of this paper are as follows:

- We design a novel CSSFAN that adopts a bottom-up pyramid-aware feature aggregation strategy combined with CSSAMs, enhancing the spatial representation of high-level features while maintaining semantic consistency.
- We develop an ARPN that dynamically adjusts the number and spatial density of proposals according to local feature complexity, addressing the uneven spatial distribution of defects.
- Extensive experiments conducted on two benchmark datasets for strip steel surface defect detection demonstrate that the proposed method consistently outperforms state-of-the-art approaches in terms of detection accuracy and robustness.

The structure of this paper is as follows: Section 2 reviews related works; Section 3 details the components of our method; Section 4 presents the experimental evaluation of the proposed method; and Section 5 concludes the paper.

## 2. Related Work

### 2.1. Strip Steel Surface Defect Detection

Many studies have proposed solutions for strip steel surface defect detection, categorized into traditional and deep-learning methods [25]. Traditional detection methods for strip steel surface defects mainly include manual inspection [26], magnetic flux leakage detection [27], and infrared detection [28]. Manual inspection relies heavily on human vision and experience, which is time-consuming, labor-intensive, and prone to subjective errors. Magnetic flux leakage detection analyzes disturbances in the magnetic field to reveal hidden or surface defects, but it often requires strict testing conditions and suffers from limited sensitivity to small flaws. Infrared detection identifies defects by capturing temperature variations caused by surface irregularities, yet its accuracy can be easily influenced by environmental factors such as heat radiation and ambient temperature. Overall, while these traditional approaches provide valuable tools for defect detection, their limited efficiency, robustness, and adaptability to complex industrial environments restrict their widespread application [29].

Deep learning-based methods have been widely applied in strip steel surface defect detection, demonstrating superior performance compared to traditional approaches [30]. For instance, Shi et al. [31] employed ConvNeXt [32] as the backbone of Faster-RCNN and incorporated the CBAM module [33] to enhance feature representation, improving detection accuracy. Chen et al. [34] designed a defect detection network that integrates deformable convolution with coordinate attention, enabling the model to capture geometric transformations and long-range dependencies for more precise and efficient detection. Huang et al. [35] proposed a lightweight method based on YOLOv8n, where the GhostNetv2 [36] module was introduced to enhance feature extraction capability while maintaining computational efficiency. In addition, Shi et al. [31] used ConvNeXt as the feature extractor for Faster-RCNN and applied the CBAM to enhance the model’s feature expression capability.

### 2.2. Multiscale Feature Fusion

Multiscale feature fusion has been widely applied in steel surface defect detection, aiming to integrate information across different resolutions so that models can simultaneously capture fine-grained local structures and high-level semantic context [37]. feature pyramid networks (FPN) [38] achieve this by constructing a top-down architecture that progressively fuses semantic-rich high-level features with detail-preserving low-level features, significantly enhancing multiscale object detection performance. Building upon FPN, PAFPN [21] introduces a bottom-up path to strengthen cross-scale feature propagation, thereby improving localization precision and robustness. To further optimize feature fusion strategies, neural architecture search FPN (NAS-FPN) [39] employs automated neural architecture search to explore the design space, generating fusion schemes that outperform manually designed pyramids. In addition, bidirectional FPN (BiFPN) [40] introduces efficient bidirectional cross-scale connections with learnable weights, enabling adaptive balancing of feature importance across levels and achieving a better trade-off between accuracy and computational efficiency. Overall, these studies demonstrate that effective multiscale feature fusion plays a critical role in enhancing the robustness and adaptability of modern object detection frameworks.

## 3. Method

### 3.1. Overall

**Figure 2:**
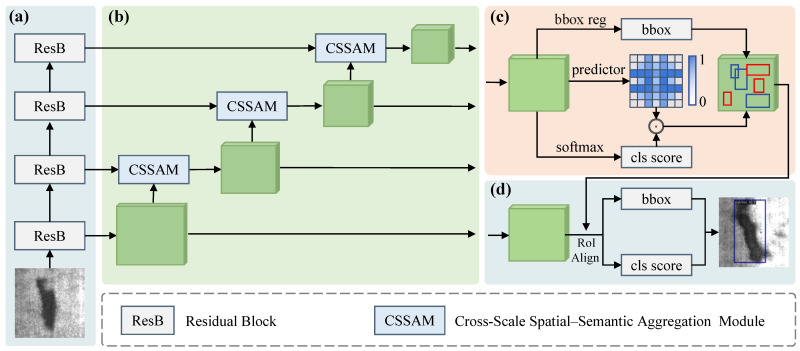
Overall Architecture of the Proposed Method. (**a**) Backbone network. (**b**) Cross-Scale Spatial–Semantic Feature Aggregation Network. (**c**) Adaptive Region Proposal Network, (**d**) Detection head.

We propose a novel strip steel surface defect detector, as illustrated in Figure 2. Our method consists of four main components: (1) the backbone network, (2) the cross-scale spatial–semantic feature aggregation network (CSSFAN), (3) the adaptive region proposal network (ARPN), and (4) the detection head.

In the feature extraction stage, the backbone network (i.e., ResNet) [41] is employed to generate hierarchical multiscale feature representations, providing a solid foundation for subsequent feature fusion and region proposal generation. To further enhance multiscale representation, we adopt a bottom-up fusion strategy and integrate cross-scale spatial–semantic aggregation modules (CSSAMs). Specifically, CSSAM establishes cross-attention mappings between high-level semantic features and their corresponding low-level feature regions, enabling bidirectional information exchange across scales. This mechanism aims to preserve semantic consistency and inject fine-grained spatial details into high-level feature maps, improving the discriminative power of fused representations. In the proposal generation stage, the adaptive region proposal network (ARPN) is introduced to address the limitations of conventional RPNs. Unlike fixed-anchor strategies, ARPN dynamically adjusts the number and distribution of anchors based on local feature complexity. This adaptive mechanism effectively reduces redundant proposals in relatively simple regions while increasing proposal density in complex regions, ensuring more balanced and efficient coverage across varying difficulty levels. Finally, the detection head refines the proposals through classification and regression, achieving fine-grained defect localization and recognition.

The primary contributions of this work lie in the proposed CSSAM and ARPN, which are elaborated in Section 3.2 and Section 3.3, respectively.

### 3.2. Cross-Scale Spatial–Semantic Aggregation Modules (CSSAM)

**Figure 3:**
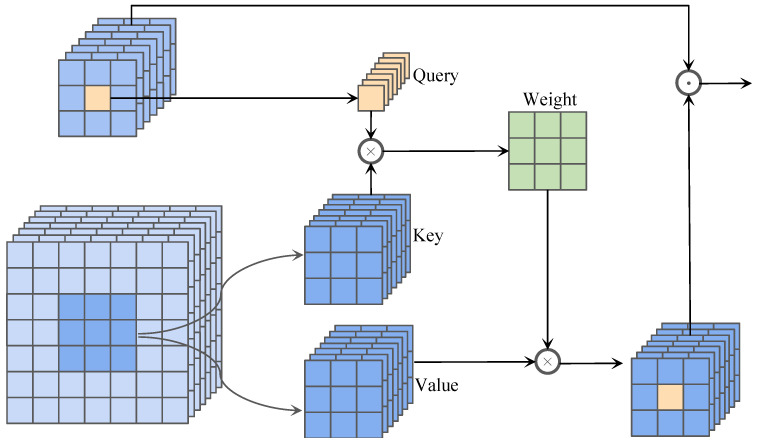
The structure of the proposed CSSAM.

As illustrated in Figure 3, we propose CSSAM to facilitate spatial information transfer between high-level semantic feature points and their corresponding low-level feature regions. This mechanism adaptively injects spatial details from low-level features into high-level semantic representations, enhancing spatial precision while maintaining semantic consistency. This design enables for more accurate defect location in complex industrial scenarios. Let the low-level feature map be denoted as $f^{i}\in R^{C\times H\times W}$ and the high-level semantic feature map as $f^{i+1}\in R^{C\times H^{\prime }\times W^{\prime }}$. The CSSAM operates through three sequential stages: (1) query construction and key–value extraction; (2) cross-scale attention computation; (3) feature reconstruction and aggregation.

#### 3.2.1. Query Construction and Key-Value Extraction

To enable efficient cross-scale feature interaction, we first sample query tokens from the high-level feature map and extract key–value pairs from the corresponding low-level feature map. This design allows the network to establish a bidirectional correspondence between fine-grained spatial information and high-level semantic representations, promoting effective multiscale information exchange.

Specifically, for a high-level feature map $f^{i+1}$ containing rich semantic cues but limited spatial resolution, we treat each spatial position as an independent query token, forming:

(1) $$Q^{i+1}=U_{k,s}\left(f^{i+1}\right)\in R^{(k^{2}C)\times L}.$$

where *C* denotes the channel dimension and $L=H^{\prime }\times W^{\prime }$ represents the total number of spatial positions. Each query token encapsulates semantic information at a specific location, serving as an anchor for cross-scale interaction.

In parallel, the corresponding low-level feature map $f^{i}$, which retains fine-grained texture and boundary information, is processed using an unfolding operator $U_{k,s}\left(\cdot \right)$ with kernel size k×k and stride *s* to partition it into a series of non-overlapping local patches:

(2) $$K^{i}=V^{i}=U*k,s{\left(f^{i}\right)}^{⊤}\in R^{L\times C},$$

Each patch preserves detailed local structural information and serves as a spatial reference during attention computation, while the transposition ${\left(\cdot \right)}^{⊤}$ aligns spatial and channel dimensions for efficient matrix operations. By aligning the high-level queries $Q^{i+1}$ with the low-level keys and values $\left(K^{i},V^{i}\right)$, the subsequent cross-attention mechanism adaptively transfers spatial details from the low-level domain to enhance high-level semantic features. This process effectively bridges the gap between spatial precision and semantic abstraction, enabling the network to accurately locate subtle and irregular defect patterns across multiple scales.

#### 3.2.2. Cross-Attention Computation

We compute cross-scale attention using the constructed query, key, and value matrices to enable semantic propagation from high-resolution to low-resolution features. The cross-scale attention is formally defined as:

(3) $$A^{i+1}=\mathrm{Softmax}\left(\frac{Q^{i+1}{\left(K^{i}\right)}^{⊤}}{\sqrt{d}}\right)V^{i},$$

where *d* denotes the embedding dimension used for normalization. The dot-product operation measures the similarity between each query and key, while the Softmax function converts this similarity map into a probabilistic attention distribution. Through this adaptive weighting mechanism, the network selectively aggregates high-resolution spatial information that is most relevant to each low-resolution position. Consequently, high-level features are enriched with fine-grained spatial cues, whereas low-level features gain enhanced semantic coherence. This process achieves a balanced representation between spatial precision and semantic abstraction, benefiting both object localization and semantic recognition.

#### 3.2.3. Feature Reconstruction and Fusion

We reconstruct and fuse the attention-enhanced representation $A^{i+1}$ with the original high-level feature $f^{i+1}$ to obtain a unified multiscale representation. The attention output $A^{i+1}$ carries spatially detailed information propagated from low-level features, serving as an effective modulation signal to refine the high-level semantics.

To integrate these complementary cues, $A^{i+1}$ is first reshaped to match the spatial structure of the original feature map and then fused via element-wise multiplication:

(4) $${\hat{f}}^{i+1}=\mathrm{Reshape}\left(A^{i+1}\right)\cdot f^{i+1}.$$

This operation injects fine-grained spatial information into the semantic representation, producing an enhanced feature map ${\hat{f}}^{i+1}$ that preserves both spatial fidelity and semantic richness.

In contrast to conventional feature addition or concatenation, the proposed multiplicative fusion adaptively modulates each spatial position according to its contextual relevance. This design facilitates more precise structural alignment and promotes smoother optimization during cross-scale fusion, ultimately yielding a more discriminative and robust representation for downstream detection and recognition tasks.

### 3.3. Adaptive Region Proposal Network (ARPN)

**Figure 4:**
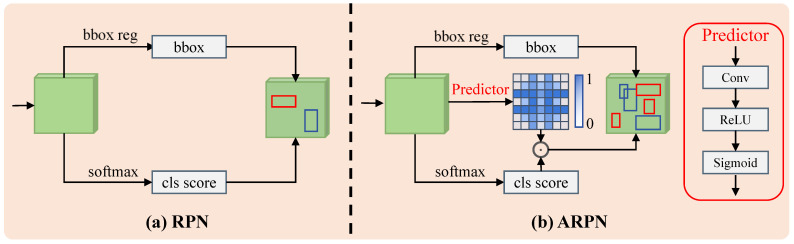
The structure of the proposed ARPN.

As shown in Figure 4a, conventional RPN generates a fixed number of anchors at each spatial location of the feature map, regardless of the local visual complexity. This design inevitably leads to redundant anchors in simple regions and insufficient coverage in complex regions with rich structures. To address this limitation, we propose an ARPN (Figure 4b) that dynamically adjusts the anchor density according to the local feature complexity, thereby improving proposal quality and reducing computational redundancy.

#### 3.3.1. Density Score Estimation

Given an input feature map ${\hat{f}}^{i}\in R^{C\times H\times W}$, a lightweight convolutional predictor is designed to estimate a *density score map* $D\in {\left(0,1\right)}^{H\times W}$, which quantifies the structural complexity of each spatial region. Intuitively, areas containing rich texture details or multiple defect cues are more likely to require denser anchor sampling for precise localization. Each element $D_{i}$ represents the local structural complexity at spatial position *i*, where a higher value implies a more intricate region that requires a finer anchor coverage. The density score is computed as:

(5) $$D_{i}=\sigma \left(W_{2}\cdot \delta \left(W_{1}*{\hat{f}}^{i}\right)\right),$$

where ∗ denotes convolution, δ(·) is the ReLU activation, and σ(·) is the Sigmoid function that normalizes responses into (0,1). The learnable parameters $W_{1}$ and $W_{2}$ are jointly optimized with the entire detection framework in an end-to-end manner, enabling the model to automatically learn an adaptive density representation consistent with the spatial distribution of surface defects.

#### 3.3.2. Adaptive Anchor Reweighting

During candidate proposal generation, the estimated density score $D_{i}$ is integrated with the classification confidence score $s_{i}$ predicted by the RPN head to produce an adaptive confidence score

(6) $${\tilde{s}}_{i}=s_{i}\cdot D_{i}.$$

This reweighting mechanism adaptively balances the importance of each anchor according to the underlying spatial complexity. Specifically, anchors located in regions with high density scores, which typically contain fine-grained textures or multiple small-scale defects, are assigned larger weights to ensure adequate proposal generation and reduce the risk of missed detections. In contrast, anchors in smooth or homogeneous regions are down-weighted, suppressing redundant or low-quality proposals. Through this dynamic reweighting strategy, the proposed method achieves an improved trade-off between recall and precision, enabling the RPN to generate more informative and spatially balanced proposals that subsequently enhance feature refinement and detection accuracy.

### 3.4. Loss Function

Our method is optimized using a composite loss function consisting of a classification term [42] and a bounding box regression term [43], expressed as

(7) $$L=L_{\mathrm{cls}}+L_{\mathrm{box}}.$$

Here, $L_{\mathrm{cls}}$ evaluates the discrepancy between predicted class probabilities and ground-truth labels, and is formulated as

(8) $$L_{\mathrm{cls}}=-g\log\left(p\right)-\left(1-g\right)\log\left(1-p\right),$$

where *p* denotes the predicted confidence score and g∈{0,1} represents the ground-truth class indicator. The second term, $L_{\mathrm{box}}$, enforces accurate localization by penalizing the deviation between the predicted bounding box and its ground truth:

(9) $$L_{\mathrm{box}}=\sum_{j\in x,y,w,h}L_{1}\left(t_{j}-{\hat{t}}_{j}\right),$$

where $t_{j}=\left(t_{x},t_{y},t_{w},t_{h}\right)$ indicates the ground-truth box and ${\hat{t}}_{j}=\left({\hat{t}}_{x},{\hat{t}}_{y},{\hat{t}}_{w},{\hat{t}}_{h}\right)$ denotes the predicted box.

## 4. Experiments

### 4.1. Experimental Setup

#### 4.1.1. Implementation Details

Our method is implemented based on the MMDetection framework [44]. All experiments are conducted on a computing platform running Ubuntu 20.04 with a single NVIDIA A100 GPU (80 GB memory). The software environment includes Python 3.9, PyTorch 1.13.1, and CUDA 11.7. Our code is available at https://github.com/hpguo1982/CSSFAN (accessed on 11 November 2025). Detailed hyperparameter settings are listed in Table 1.

#### 4.1.2. Datasets

To validate the effectiveness of the proposed method, we conduct extensive experiments on two challenging steel surface defect datasets: NEU-DET [45,46,47] and GC10-DET [48].

NEU-DET Dataset: The NEU-DET dataset, released by Northeastern University, is one of the most representative benchmark datasets for industrial surface defect detection. It consists of 1800 grayscale images collected from hot-rolled steel strips, covering six typical types of surface defects commonly encountered in industrial production: scratch (Sc), patch (Pa), inclusion (In), rolled-in scale (Rs), pitted surface (Ps), and crazing (Cr). Each category contains 300 images with a resolution of 200×200 pixels. Due to its balanced distribution across defect types and its high relevance to real-world inspection scenarios, NEU-DET has become a widely adopted dataset for evaluating the robustness, recognition accuracy, and generalization capability of defect detection models.

GC10-DET Dataset: The GC10-DET dataset was collected from real-world hot-rolled steel production lines and contains 2294 images with varying resolutions. It covers ten representative categories of surface defects, namely punch (Pu), welding line (Wl), crescent gap (Cg), water spot (Ws), oil spot (Os), silk spot (Ss), inclusion (In), roll pit (Rp), crease (Cr), and waist fold (Wf). Each category contains dozens to hundreds of samples, exhibiting a wide range of variations in scale, shape, texture, and intensity. Compared with NEU-DET, GC10-DET is more challenging due to its higher intra-class diversity, complex backgrounds, and inter-class similarities. As such, it provides a more realistic benchmark for evaluating the robustness, adaptability, and generalization ability of defect detection algorithms in industrial inspection tasks.

#### 4.1.3. Evaluation Metrics

We evaluate the detection performance following the COCO evaluation protocol [49]. Specifically, we report ${\mathrm{AP}}_{50}$ (IoU threshold = 0.5), ${\mathrm{AP}}_{75}$ (IoU threshold = 0.75), and the overall AP averaged over IoU thresholds from 0.5 to 0.95. Furthermore, scale-aware metrics are also included, i.e., ${\mathrm{AP}}_{S}$, ${\mathrm{AP}}_{M}$, and ${\mathrm{AP}}_{L}$, which correspond to objects of small ($\mathrm{area}<32^{2}$), medium ($32^{2}\leq \mathrm{area}<96^{2}$), and large sizes ($\mathrm{area}\geq 96^{2}$), respectively.

### 4.2. Quantitative Comparison

We compare our method with fifteen state-of-the-art methods on the NEU-DET and GC10-DET datasets, including SSD300 [50], TOOD [51], Faster-RCNN [52], DH-RCNN [53], Cascade-RCNN [54], Dynamic-RCNN [55], Grid-RCNN [56], Libra-RCNN [57], Sparse-RCNN [58], YOLOv9 [59], YOLOv10 [60], DETR [61], RT-DETR [62], CA-Autoassign [24] and STD2 [20], the results are shown in Table 2 and Table 3.

#### 4.2.1. Quantitative Comparison on NEU-DET Dataset

As reported in Table 2, our proposed method demonstrates superior performance on the NEU-DET dataset across multiple evaluation metrics. Specifically, it attains the highest overall AP of 45.1%, along with the best scores in AP_50_ (81.1%), AP_75_ (46.5%), AP_*S*_ (43.3%), and AP_*L*_ (56.9%), indicating its strong capability in achieving both coarse and fine-grained localization for small and large defects. Although its AP_*M*_ ranks third, slightly below Cascade-RCNN and DETR, the performance gap is marginal, showing the competitive balance of CA-RCNN across object scales. In terms of category-level evaluation, our method achieves the highest AP values on four representative defect types, namely Cr (53.6%), In (86.8%), Pa (95.1%), and Ps (93.2%). For the remaining Rs and Sc categories, our method also delivers competitive results, ranking closely to the best-performing methods.

#### 4.2.2. Quantitative Comparison on GC10-DET Dataset

As shown in Table 3, our method demonstrates consistently superior performance on the GC10-DET dataset. our method achieves the highest scores in overall AP (35.9%), AP_50_ (72.3%), AP_*S*_ (14.1%), and AP_*L*_ (40.1%), highlighting its effectiveness in accurately detecting defects across varying object sizes, particularly small-scale and large-scale defects. In addition, it delivers competitive results on AP_75_ and AP_*M*_, further illustrating the robustness of the proposed framework across different IoU thresholds and object scales. At the category level, our method outperforms other approaches in several representative defect types, achieving the highest AP values for Pu (97.4%), Wl (98.3%), Ss (67.8%), In (43.7%), Rp (50.6%), and Cr (56.5%). Moreover, our method maintains strong competitiveness on other categories, such as Ws (78.9%) and Wf (72.8%), which involve challenging backgrounds and complex textures.

### 4.3. Visual Comparison

**Figure 5:**
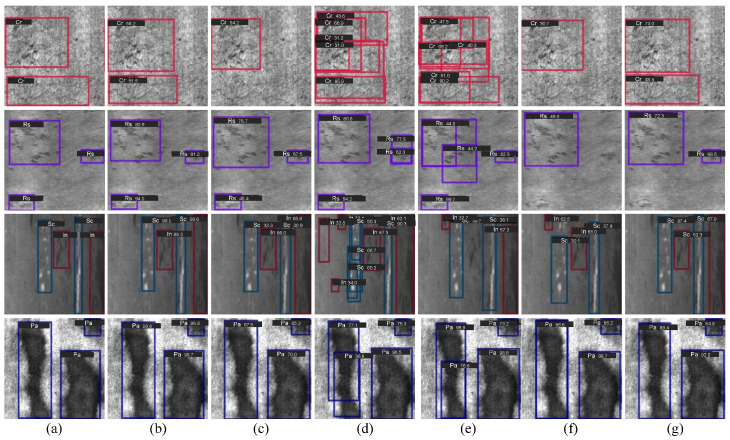
Visual Comparison on the NEU-DET Dataset. (**a**) GT. (**b**) Ours. (**c**) TOOD. (**d**) Faster-RCNN. (**e**) Cascade-RCNN. (**f**) RT-DETR. (**g**) STD2.

**Figure 6:**
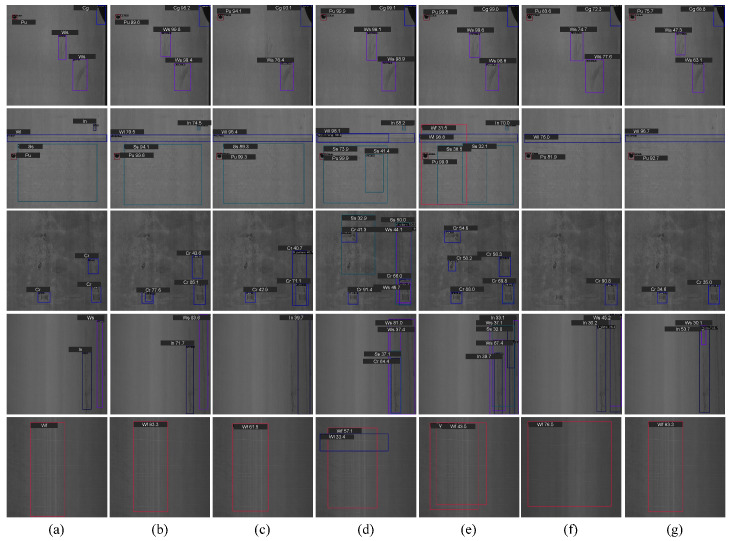
Visual Comparison on the GC10-DET Dataset. (**a**) GT. (**b**) Ours. (**c**) TOOD. (**d**) Faster-RCNN. (**e**) Cascade-RCNN. (**f**) RT-DETR. (**g**) STD2.

We present the visualization results of six methods, i.e., Tood [51], Faster-RCNN [52], Cascade-RCNN [54], RT-DETR [62], STD2 [20], and our method, on the NEU-DET and GC10-DET datasets, as illustrated in Figure 5 and Figure 6, respectively.

#### 4.3.1. Visual Comparison on NEU-DET

As shown in Figure 5, the proposed method (column 2) exhibits the most favorable detection performance on the NEU-DET dataset, with results closely aligned with the ground truth. Specifically, for low-contrast defects (row 1), Faster-RCNN and Cascade-RCNN erroneously classify background regions as defect objects, while TOOD and RT-DETR fail to detect certain instances. For point-like defects (row 2), RT-DETR and STD2 both suffer from false detections. In cases of elongated or small defects (row 3), Faster-RCNN generates redundant bounding boxes, whereas Cascade-RCNN fails to detect some defect instances. For defects with diverse shapes (row 4), Faster-RCNN and Cascade-RCNN do not accurately localize the targets. In contrast, the proposed method effectively suppresses background noise, mitigates misidentification, and demonstrates superior precision and robustness across various defect types.

#### 4.3.2. Visual Comparison on GC10-DET

As illustrated in Figure 6, we present the visual comparison between our method and other state-of-the-art detectors on the GC10-DET dataset. From Figure 6, it can be observed that in cases of multiple defect detection (rows 1 and 2), TOOD suffers from false negatives (i.e., misclassifying defects as background), while Faster-RCNN and Cascade-RCNN exhibit false positives (i.e., misclassifying background as defects). For low-contrast defects (rows 3 and 4), TOOD, Faster-RCNN, and Cascade-RCNN all produce false positives, whereas RT-DETR and STD2 show false negatives. In the last row of defects, both Faster-RCNN and Cascade-RCNN generate false positives. In contrast, our method effectively suppresses these errors and achieves precise localization. Furthermore, although all methods can successfully detect defects in the last two rows, our method demonstrates superior stability and a clear advantage when addressing low-contrast and complex defect scenarios.

### 4.4. Ablation Experiments

To evaluate the effectiveness of each component in our method, we progressively incorporate CSSFAN, and ARPN into the baseline model, the ablation results are shown in Table 4.

From Table 4, the introduction of CSSFAN improves AP from 43.4% to 44.5%, with consistent gains observed in AP_50_ and AP_75_, indicating that CSSFAN effectively enhances multiscale feature representation. Incorporating ARPN alone yields an AP of 43.9%, and further contributes to the detection of small and large objects, as reflected by improvements in AP_*S*_ (42.6%) and AP_*L*_ (53.6%). When both CSSFAN and ARPN are simultaneously integrated, the proposed framework achieves the highest overall performance with an AP of 45.1%, along with consistent improvements across all evaluation metrics, including AP_50_, AP_75_, and object scales. These results confirm the complementary nature of CSSFAN and ARPN, and validate the effectiveness of the proposed method.

### 4.5. Effectiveness of CSSFAN

To further validate the effectiveness of CSSFAN, we conduct comparative experiments with five mainstream feature fusion networks (FPN [38], NAS-FPN [39], PAFPN [21], BiFPN [40], and AFPN [63]) on the NEU-DET dataset. The comparison results are shown in Table 5.

From Table 5, our CSSFAN achieves the best performance across almost all evaluation metrics. Specifically, our method attains the highest AP of 45.1%, outperforming the second-best PAFPN (44.0%) by 1.1 percentage points. In terms of AP_50_ and AP_75_, CSSFAN reaches 81.1% and 46.5%, respectively, indicating stronger localization accuracy and robustness. Furthermore, CSSFAN demonstrates consistent improvements across different object scales, achieving the best AP_S_, and AP_L_ scores of 43.3%, and 56.9%, respectively. These results clearly verify that the proposed cross-scale and semantic feature aggregation strategy in CSSFAN effectively enhances multiscale feature representation and detection precision compared to conventional and state-of-the-art feature fusion architectures.

### 4.6. Generalization Experiments

To further validate the effectiveness of the proposed method, we conduct generalization experiments on the resistance spot welding defect (RSW-D) dataset [64]. The RSW-D dataset comprises 4134 images, covering seven defect categories: Normal (No), Edge (Ed), Copper-adhesion (Ca), Overlap (Ov), Mutilation (Mu), Splash (Sp), and Twist (Tw). Table 6 shows the corresponding results on the RSW-D datasets.

As shown in Table 6, the proposed method consistently surpasses all compared methods in terms of AP, AP_50_, AP_75_, AP_*M*_, and AP_*L*_, achieving the highest scores of 70.9%, 97.2%, 87.6%, 67.7%, and 71.8%, respectively, while ranking second on AP_*S*_. At the category level, our method achieves the best AP_50_ results in Wl (99.0%), Cg (98.0%), Os (97.6%), Ss (96.6%), and In (97.0%), and obtains comparable performance in Pu and Ws. Overall, these results clearly verify that our method achieves superior generalization and detection stability across both challenging defect scales and heterogeneous categories, further validating its effectiveness and robustness across diverse defect types.

## 5. Conclusions

In this paper, we proposed a novel Cross-Scale Spatial–Semantic Feature Aggregation Network (CSSFAN) for steel surface defect detection, addressing the challenges posed by diverse defect scales and uneven spatial distributions. The proposed network integrates multi-level features through a bottom-up aggregation strategy, where the Cross-Scale Spatial–Semantic Aggregation Module (CSSAM) adaptively fuses low-level spatial details with high-level semantic information, enhancing feature completeness and strengthening the representation of subtle and irregular defect patterns. To further alleviate the problem of spatial imbalance, an Adaptive Region Proposal Network (ARPN) was introduced to dynamically adjust the number and distribution of proposals according to local feature complexity, allowing the network to focus on defect-prone regions while suppressing redundant proposals in homogeneous areas. Extensive experiments conducted on two benchmark strip steel defect datasets demonstrate that CSSFAN consistently outperforms existing detection methods in both accuracy and robustness. These results confirm the effectiveness of the proposed cross-scale aggregation and adaptive proposal mechanisms, offering a promising direction for high-precision industrial surface defect inspection. 

## Figures and Tables

**Table 1:** Hyperparameter setting of CA-RCNN.

Hyperparameters	Learning Rate	Weight Decay	Momentum Coefficien	Optimizer	Batch Size	Train:Test	Epoch
Value	0.008	0.0001	0.9	SGD	2	8:2	36

**Table 2:** Quantitative comparison with 15 SOTA methods on the NEU-DET dataset.

Methods	AP	AP_50_	AP_75_	AP_*S*_	AP_*M*_	AP_*L*_	Cr	In	Pa	Ps	Rs	Sc
SSD300 [50]	39.7	73.7	34.4	33.8	33.3	51.2	48.8	82.7	94.2	85.9	63.8	66.9
TOOD [51]	41.2	76.7	38.7	36.7	31.9	52.5	38.6	86.4	90.9	90.5	65.0	88.8
Faster-RCNN [52]	43.4	78.9	43.2	41.7	37.7	51.9	44.7	85.4	93.2	92.5	65.7	**95.3**
DH-RCNN [53]	36.7	75.0	33.5	40.3	29.1	40.7	40.4	79.4	87.6	88.5	63.0	91.2
Cascade-RCNN [54]	44.0	79.3	44.0	41.6	39.8	53.9	47.0	84.5	91.7	89.5	**66.0**	95.3
Dynamic-RCNN [55]	40.5	76.5	38.9	41.1	34.8	49.9	43.7	83.2	93.0	89.2	57.2	92.7
Grid-RCNN [56]	41.8	75.9	41.4	38.7	34.0	53.3	40.1	85.7	92.5	89.9	56.2	90.8
Libra-RCNN [57]	40.0	74.6	38.2	42.5	35.4	44.2	40.1	84.5	90.7	83.9	59.5	88.9
Sparse-RCNN [58]	39.7	71.3	39.6	35.9	33.1	47.9	37.4	80.8	89.1	90.1	49.3	81.1
YOLOv9 [59]	42.5	76.0	41.9	43.1	36.2	50.1	44.6	84.7	92.5	87.8	52.7	92.8
YOLOv10 [60]	41.3	77.3	41.5	42.6	38.2	49.6	45.4	82.4	91.7	90.4	62.9	90.8
DETR [61]	44.1	73.2	44.1	34.5	41.8	52.3	39.7	81.1	88.2	78.0	55.4	94.8
RT-DETR [62]	44.5	75.5	44.4	37.6	39.0	53.7	43.6	86.3	91.8	83.0	58.6	90.0
CA-Autoassign [24]	39.5	77.0	41.5	34.6	24.0	45.4	44.4	84.1	90.4	83.4	65.8	93.6
STD2 [20]	43.1	80.4	41.7	36.7	38.7	53.1	52.9	85.3	94.1	93.1	64.0	93.1
Ours	**45.1**	**81.1**	**46.5**	**43.3**	39.2	**56.9**	**53.6**	**86.8**	**95.1**	**93.2**	64.8	93.3

The bold text indicates the best result among all methods.

**Table 3:** Quantitative comparison with 15 SOTA methods on the GC10-DET dataset.

Methods	AP	AP_50_	AP_75_	AP_*S*_	AP_*M*_	AP_*L*_	Pu	Wl	Cg	Ws	Os	Ss	In	Rp	Cr	Wf
SSD300 [50]	27.8	58.1	20.1	8.7	24.5	29.4	93.6	76.4	90.8	73.5	55.1	60.0	37.3	14.5	44.8	35.4
TOOD [51]	34.5	65.2	31.3	12.6	28.9	39.0	93.2	79.8	90.1	76.5	66.0	59.7	26.2	33.5	55.9	70.7
Faster-RCNN [52]	34.2	68.0	29.6	10.2	30.5	34.2	95.7	95.2	90.5	76.2	65.0	63.1	37.6	48.8	37.2	70.5
DH-RCNN [53]	30.6	65.8	26.4	9.9	29.4	30.4	96.9	79.4	84.0	**80.8**	70.2	67.1	34.9	33.3	39.0	70.2
Cascade-RCNN [54]	34.8	69.9	30.9	11.9	30.0	38.7	94.5	96.9	88.6	75.0	**71.3**	63.7	38.0	46.5	50.4	**73.8**
Dynamic-RCNN [55]	30.3	62.2	23.9	10.6	29.9	30.6	97.2	96.4	86.5	69.3	67.8	57.9	31.4	25.9	31.0	58.5
Grid-RCNN [56]	30.1	63.1	24.2	11.3	28.7	30.9	96.3	92.3	85.6	67.3	69.4	54.2	30.5	32.3	37.2	68.0
Libra-RCNN [57]	27.5	57.9	22.1	12.9	28.9	26.6	**97.4**	93.6	88.2	67.4	65.3	54.8	17.5	18.2	27.0	49.9
Sparse-RCNN [58]	32.3	65.8	30.0	8.1	27.7	34.7	96.9	98.2	87.4	71.0	67.2	62.4	29.0	30.1	42.4	72.4
YOLOv9 [59]	33.6	67.6	31.5	14.2	29.7	35.9	91.5	82.8	**92.9**	79.6	70.0	66.8	25.7	41.1	56.1	70.8
YOLOv10 [60]	32.9	64.2	31.0	13.5	30.6	34.7	93.2	79.8	96.1	77.5	65.0	61.7	19.2	30.5	55.9	73.7
DETR [61]	31.7	68.8	30.1	12.1	27.0	36.9	95.8	90.3	90.9	80.4	55.4	60.1	43.0	43.3	55.4	71.5
RT-DETR [62]	30.1	69.7	22.3	11.6	25.5	35.0	96.7	88.7	92.1	80.4	62.3	65.2	41.5	45.3	55.0	70.1
CA-Autoassign [24]	24.3	62.6	27.5	9.7	22.7	32.9	95.9	77.7	92.7	70.5	62.0	61.7	35.3	31.8	32.5	65.7
STD2 [20]	35.0	71.0	29.8	12.1	32.0	38.3	95.6	96.5	87.0	77.7	70.8	63.9	42.3	48.5	56.4	70.0
Ours	**35.9**	**72.3**	30.0	**14.1**	31.5	**40.1**	**97.4**	**98.3**	88.6	78.9	67.9	**67.8**	**43.7**	**50.6**	**56.5**	72.8

The bold text indicates the best result among all methods.

**Table 4:** Ablation study results on the NEU-DET datasets.

Methods	AP	AP_50_	AP_75_	AP_*S*_	AP_*M*_	AP_*L*_
Baseline	43.4	78.9	43.2	41.7	37.7	51.9
Baseline + CSSFAN	44.5	80.0	45.4	42.4	38.1	54.2
Baseline + ARPN	43.9	79.8	44.3	42.6	38.9	53.6
Baseline + CSSFAN + ARPN	45.1	81.1	46.5	43.3	39.2	56.9

**Table 5:** Comparison results between CSSFAN and five mainstream feature fusion networks on the NEU-DET dataset.

Methods	AP	AP_50_	AP_75_	AP_*S*_	AP_*M*_	AP_*L*_
FPN [38]	43.4	78.9	43.2	41.7	37.7	51.9
NAS-FPN [39]	41.7	77.1	42.4	40.2	34.9	49.5
PAFPN [21]	44.0	79.3	44.9	42.6	**39.9**	54.3
BiFPN [40]	42.9	78.5	**47.1**	42.3	38.7	53.6
AFPN [63]	42.6	76.3	40.2	40.0	35.1	50.3
CSSFAN (Ours)	**45.1**	**81.1**	46.5	**43.3**	39.2	**56.9**

The bold text indicates the best result among all methods.

**Table 6:** Generalization experiments on the RSW-D dataset.

Methods	AP	AP_50_	AP_75_	AP_*S*_	AP_*M*_	AP_*L*_	Pu	Wl	Cg	Ws	Os	Ss	In
SSD300 [50]	66.0	95.5	79.4	39.5	55.7	67.0	95.7	98.3	96.8	93.8	**97.6**	94.7	91.7
TOOD [51]	70.5	95.9	86.2	**48.3**	64.8	71.5	**97.5**	98.4	95.0	**96.4**	98.0	96.1	90.1
Faster-RCNN [52]	68.3	95.7	82.4	41.7	63.1	69.2	95.4	96.9	96.7	95.4	95.5	95.5	93.6
DH-RCNN [53]	66.9	94.2	83.0	43.1	62.9	67.3	94.2	95.0	95.0	93.8	94.7	92.6	94.5
Cascade-RCNN [54]	69.9	96.5	85.4	43.2	67.9	70.6	95.5	98.9	96.8	96.0	97.4	95.6	95.0
Dynamic-RCNN [55]	62.1	91.8	80.0	40.6	57.5	64.1	91.1	93.3	92.6	90.4	92.5	91.3	91.4
Grid-RCNN [56]	63.7	93.8	81.5	41.6	60.3	65.9	95.2	96.4	93.3	94.7	95.2	94.1	88.1
Libra-RCNN [57]	60.6	90.7	76.3	42.1	51.5	62.5	91.7	93.4	91.9	90.3	92.5	89.5	96.4
Sparse-RCNN [58]	62.6	92.8	81.1	40.3	56.4	64.9	92.3	94.3	93.5	91.3	93.8	92.7	92.4
YOLOv9 [59]	65.5	94.5	70.6	44.1	57.7	61.4	94.8	97.2	95.7	92.8	96.7	93.6	90.8
YOLOv10 [60]	66.1	94.7	80.1	40.6	61.9	65.2	94.5	95.8	95.6	94.5	95.3	94.7	92.6
DETR [61]	64.5	92.9	83.1	46.3	61.5	68.2	94.3	95.2	92.2	93.5	95.1	93.1	87.0
RT-DETR [62]	67.2	95.4	81.8	45.5	54.1	68.3	96.7	98.5	95.7	95.2	96.0	94.7	91.1
CA-Autoassign [24]	67.6	95.7	81.4	47.3	56.8	68.5	96.5	98.6	96.8	95.4	96.3	94.9	91.2
STD2 [20]	68.1	96.8	84.0	44.6	62.5	69.1	96.4	98.0	97.8	95.8	97.5	96.1	96.0
Ours	**70.9**	**97.2**	**87.6**	47.6	**67.7**	**71.8**	96.1	**99.0**	**98.0**	96.2	**97.6**	**96.6**	**97.0**

The bold text indicates the best result among all methods.

## Data Availability

The original contributions presented in this study are included in the article. Further inquiries can be directed to the corresponding authors.
